# Olive Biophenols Reduces Alzheimer’s Pathology in SH-SY5Y Cells and APPswe Mice

**DOI:** 10.3390/ijms20010125

**Published:** 2018-12-30

**Authors:** Syed Haris Omar, Christopher J. Scott, Adam S. Hamlin, Hassan K. Obied

**Affiliations:** 1 School of Biomedical Sciences, Faculty of Sciences and Graham Centre for Agricultural Innovation, Charles Sturt University, Wagga Wagga, NSW 2678, Australia; chscott@csu.edu.au (C.J.S.); obiedhk@gmail.com (H.K.O.); 2 School of Science & Technology, University of New England, Armidale, NSW 2351, Australia; ahamlin@une.edu.au

**Keywords:** Alzheimer’s disease, amyloid beta, SH-SY5Y cells, olive biophenols, oleuropein, verbascoside, rutin

## Abstract

Alzheimer’s disease (AD) is a major neurodegenerative disease, associated with the hallmark proteinacious constituent called amyloid beta (Aβ) of senile plaques. Moreover, it is already established that metals (particularly copper, zinc and iron) have a key role in the pathogenesis of AD. In order to reduce the Aβ plaque burden and overcome the side effects from the synthetic inhibitors, the current study was designed to focus on direct inhibition of with or without metal-induced Aβ fibril formation and aggregation by using olive biophenols. Exposure of neuroblastoma (SH-SY5Y) cells with Aβ_42_ resulted in decrease of cell viability and morphological changes might be due to severe increase in the reactive oxygen species (ROS). The pre-treated SH-SY5Y cells with olive biophenols were able to attenuate cell death caused by Aβ_42_, copper- Aβ_42_, and [laevodihydroxyphenylalanine (l-DOPA)] l-DOPA-Aβ_42_-induced toxicity after 24 h of treatment. Oleuropein, verbascoside and rutin were the major anti-amyloidogenic compounds. Transgenic mice (APPswe/PS1dE9) received 50 mg/kg of oleuropein containing olive leaf extracts (OLE) or control diet from 7 to 23 weeks of age. Treatment mice (OLE) were showed significantly reduced amyloid plaque deposition (*p* < 0.001) in cortex and hippocampus as compared to control mice. Our findings provide a basis for considering natural and low cost biophenols from olive as a promising candidate drug against AD. Further studies warrant to validate and determine the anti-amyloid mechanism, bioavailability as well as permeability of olive biophenols against blood brain barrier in AD.

## 1. Introduction

Alzheimer’s disease (AD) is associated with an abnormal accumulation and clearance of proteins known as amyloid beta (Aβ) and tau in the brain. In healthy individuals, the production and clearance of Aβ are rapid, estimated at ~7.6% and 8.3% respectively, of the total volume of Aβ produced per hour [1]. The discovery of Aβ and its accumulation in brain resulted in the formulation of the “*Amyloid Cascade Hypothesis*” which states that the deposition of Aβ subsequently leads to the formation of neurofibrillary tangles, neuronal cell death and dementia [2]. Studies have showed that the Aβ_42_ fragments are more aggregation prone than the more prevalent but less active Aβ_40_ fragment and an increase in the cerebrospinal fluid (CSF) Aβ_42_:Aβ_40_ ratio is also associated with increased neurotoxicity [3]. The brain requires metal ions for a number of important activities including the neuronal activity within the synapses and metalloproteins cellular processes [4]. In contrast, the growing evidences suggested that metals such as copper (Cu), zinc (Zn) and iron (Fe), concentrate in and around the amyloid plaques, play an important role in the pathogenesis of AD [5]. Copper enhance amyloid precursor protein (APP) dimerization and increase in extracellular Aβ_42_ release [6]. Both APP and Aβ have strong Cu-reductase activity, generating Cu^+^ from Cu^2+^ followed by the production of hydrogen peroxide as by-product [7]. However, Cu^+^ is a potent mediator of the highly reactive hydroxyl radical (OH^•^) and APP or Aβ-associated Cu^+^ may contribute to the elevated oxidative stress characteristic of AD brain [8]. The higher affinity of copper ions with Aβ_42_ than Aβ_40_, suggested its roles as inducer in Aβ aggregation [9]. Moreover, studies have shown that the long term administration of l-DOPA could lead to neurotoxicity and the inflammatory response in the brain, along with the imbalance in biothiols metabolism and plasma total homocysteine [10,11], a well-established independent risk factor for AD [12]. A few studies have also reported that the elevated l-DOPA levels result in an indirect increase in phosphorylation of tau protein [13]. Due to the aggregation prone behaviour and potent neurotoxicity of amyloid fibrils in the brain, the strategy of inhibiting Aβ_42_ aggregation has emerged as one of the valid disease modifying therapy for AD [14].

The limited available synthetic drugs used in AD, and none of the synthetic regimens to date are free from side effects, causing serious interactions and limitations. In the past decade, a substantial number of successful experimental (*in vitro* and *in vivo*) and clinical studies have been conducted to evaluate the consumption of different sources of plant biophenols in the prevention and treatment of AD [15,16]. Substantial evidences have been documented and favouring the different sources of plant biophenols either individual or extracts including caffeic acid, catechins, curcumin, luteolin, morin, quercetin, resveratrol and tannic acid were inhibited *in vitro* and *in vivo* amyloid formation [15,17]. 

The olive tree (*Olea europaea* L.) is well known for edible oil crop worldwide having great commercial value and health benefits are attributed to the oil composition (monounsaturated fatty acid) and the presence of minor components known as biophenols such as oleuropein, hydroxytyrosol, verbascoside and oleocanthal [16,18,19]. Recently, we have identified the phenolic constituents of commercial extracts and reported the *in vitro* antioxidant activities of the individual standard olive biophenols and the commercial extract (olive leaf extracts, OLE; olive fruit extracts, OFE; hydroxytyrosol extreme, HTE; and olivenol plus, OLP) biophenols against free radical and metal induced toxicity in SH-SY5Y cells [20]. In addition, we have reported that olive biophenols inhibited the enzymes including prime amyloid beta (Aβ) producing enzyme (β-secretase: BACE-1) and disease progression enzymes including acetylcholinesterase (AChE), butyrylcholinesterase (BChE), histone deacetylase (HDAC), and tyrosinase along with the catecholamine l-DOPA, which are involved in the pathogenesis AD [21].

To the best of our knowledge, no study has examined the direct Aβ_42_ inhibitory activity of different components of major olive biophenols as an individual or extracts. The present study is designed to focus on the in situ or *in vitro* inhibition of the Aβ fibrils formation and aggregation in neuroblastoma (SH-SY5Y) cells along with or without copper and l-DOPA as toxicity inducers through olive biophenols including non-flavonoids biophenols [caffeic acid (CA), hydroxytyrosol (HT), oleuropein (OL) and verbascoside (VB)], flavonoids biophenols [luteolin (LU), quercetin (QU) and rutin (RU)] and commercially available supplements [olive extracts olive leaf extracts (OLE), olive fruit extracts (OFE), hydroxytyrosol extreme (HTE) and olivenol plus (OLP)]. Furthermore, learning memory assessment, Aβ burden and biochemical parameters were investigated in the APPswe/PS1dE9 double transgenic mice model of AD after olive biophenols (olive leaf extract) administration.

## 2. Results

### 2.1. The Effect of Olive Biophenols on Aβ_42_ Aggregation (TEM)

**Figure 1:**
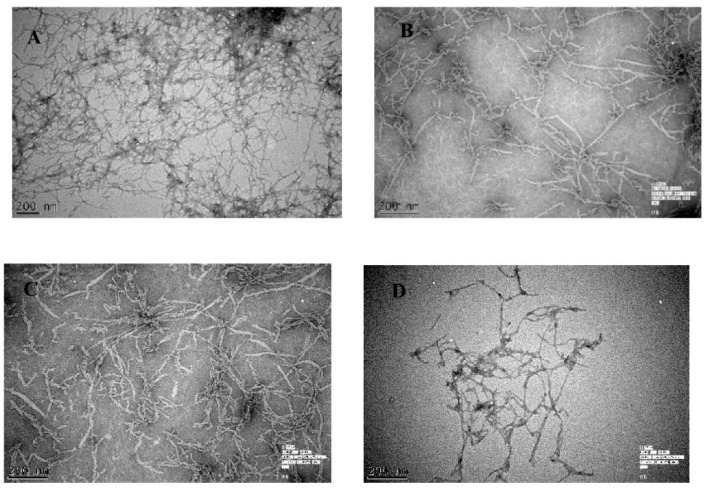
The inhibition of Aβ_42_ (20 µM) fibrils was monitored by transmission electron microscope (TEM) using ThT fluorescence in the (**A**) absence of biophenols, and presence of (**B**) oleuropein (OL) (**C**) quercetin (QU) and (**D**) olive leaf extract (OLE).

In the absence of olive biophenols, Aβ_42_ fibrils showed a typical morphology, characterized by long, straight and dense fibrils forming a brief network and analysed by TEM (Figure 1A). The incubation of olive biophenol OL (≥200 µM) with the formed Aβ_42_ fibrils cause a significant reduction in both the size and number of fibrils (Figure 1B). However, Aβ_42_ incubated with biophenol QU (≥200 µM), revealed a moderate reduction in fibril formation with the attached biophenol QU to the fibrillar species (Figure 1C). The extract olive biophenol, OLE incubation with Aβ_42_ fibrils revealed a significant reduction in both the aggregate size and occurrence (Figure 1D), with the dominant species appearing to be broken particles of fibril approximately 10 nm in diameter. A few studies [22,23] have been shown the inhibitory activity of biophenols against Aβ fibrillization and aggregation. Our studies showed that olive biophenols have also potential to inhibit the Aβ aggregation, which may protect against the AD.

### 2.2. Aβ_42_ Fibril Inhibition by Olive Biophenols (ThT Fluorometric Assay)

**Figure 2:**
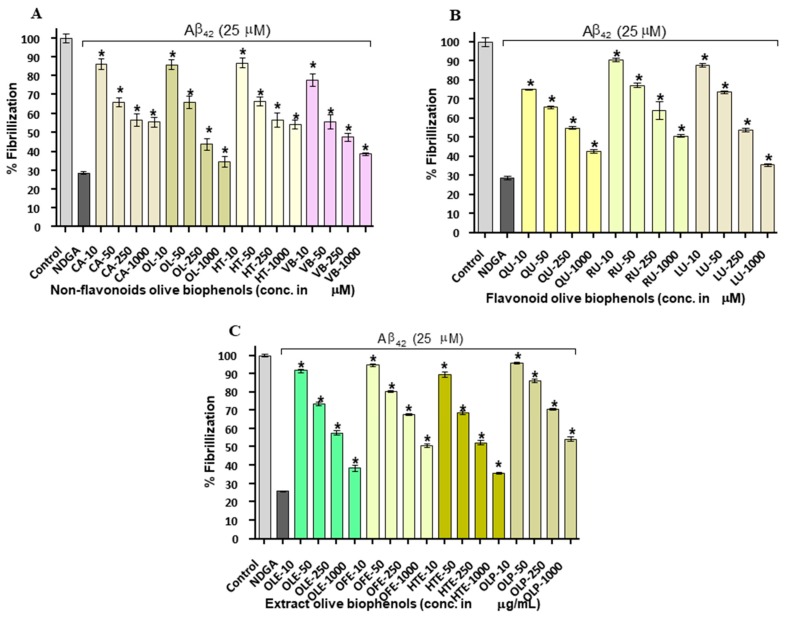
Thioflavin-T assay: Inhibition of Aβ_42_ fibrils by olive biophenols: (**A**) Non-flavonoids olive biophenols, (**B**) flavonoids olive biophenols and (**C**) extracts olive biophenols. Control: Aβ_42_ without biophenols. Nordihydroguaiaretic acid (NDGA) used as reference inhibitor. CA: caffeic acid, OL: oleuropein, HT: hydroxytyrosol, VB: verbascoside, QU: quercetin, RU: rutin, LU: luteolin, OLE: olive leaf extract, OFE: olive fruit extract, HTE: hydroxytyrosol extreme, OLP: olivenol plus. The results were mean ± S.D. analysed by one-way ANOVA (*Tukey*’s test), * *p* < 0.001 vs. negative control (NDGA).

Olive biophenols led to a concentration-dependent decrease in apparent ThT fluorescence, which on its own suggested the efficient concentration-dependent inhibition of Aβ_42_ fibrils formation in a cell free system (Table 1). The reference inhibitor NDGA showed 70% of inhibition and having an IC_50_ of 15.4 µM against the Aβ_42_ fibrillization. The non-flavonoid olive biophenols, VB and OL shared almost equal inhibitory potential of 61% (IC_50_: 22.6 µM) and 61% (IC_50_: 22.9 µM) against Aβ_42_ fibrillization (Figure 2A).

**Figure 3:**
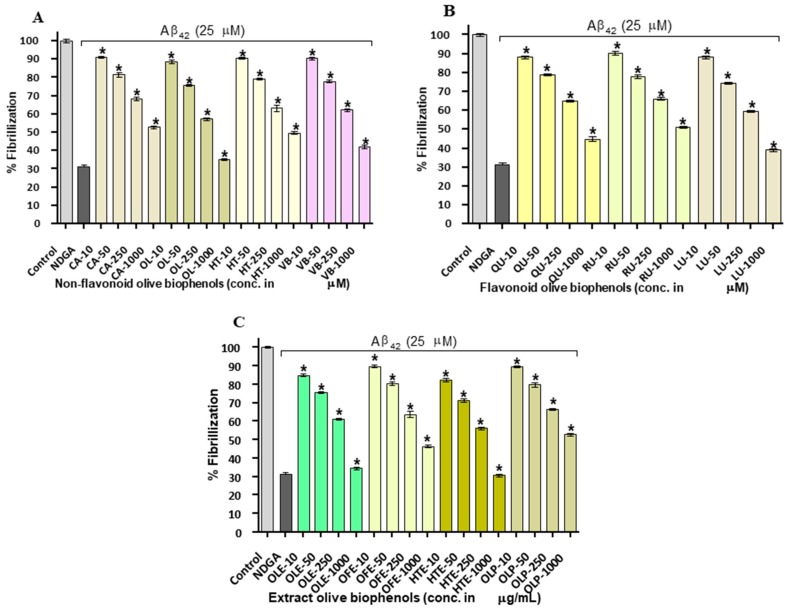
Congo red assay: Inhibition of Aβ_42_ fibrils by olive biophenols: (**A**) Non-flavonoids olive biophenols, (**B**) flavonoids olive biophenols and (**C**) extracts olive biophenols. Control: Aβ_42_ without biophenols. Nordihydroguaiaretic acid (NDGA) used as reference inhibitor. CA: caffeic acid, OL: oleuropein, HT: hydroxytyrosol, VB: verbascoside, QU: quercetin, RU: rutin, LU: luteolin, OLE: olive leaf extract, OFE: olive fruit extract, HTE: hydroxytyrosol extreme, OLP: olivenol plus. The results were mean ± S.D. analysed by one-way ANOVA (*Tukey*’s test), * *p* < 0.001 vs. negative control (NDGA).

In contrast, CA and HT were showed fewer inhibition of 46% and 45% at the maximum used concentration in the study and unable to achieve IC_50_ value (Figure 2A). The flavonoid biophenols (Figure 3B), LU showed the higher inhibition of 64% (IC_50_: 36.9 µM) than QU of 57% (IC_50_: 45.9 µM). However, RU showed the least inhibition of 49% and unable to reach the IC_50_ concentration (Figure 2B). Among the investigated biophenols-rich olive extracts (Figure 2B), HTE showed the highest inhibitory activity of 64% (IC_50_: 30.4 µg/mL) followed by OLE having 60% (IC_50_: 45 µg/mL) and OFE of 50% (IC_50_: 95.9 µg/mL). In contrast, OLP showed the least activity of 45% among extracts and unable to reach the IC_50_ concentration (Figure 2C).

A number of studies have shown that the biophenols are one of the most actively investigated categories of potential amyloid inhibitors including curcumin [24], epigallocatechin gallate [25], resveratrol [26], quercetin [27], rutin [28] and luteolin [22]. In terms of order of the potency (IC_50_), our results showed that VB > OL > LU > QR and HTE > OLE > OFE respectively. The exact mechanism of amyloid inhibition by olive biophenols is still not fully understood. On the basis of earlier proposed mechanism [29], we may suggest that the number of hydroxyl groups and their positions on biophenols structure is important for amyloid β-sheet interaction and stabilization of the inhibition and protein complex. However, researchers are still trying to understand the molecular link between phenol positional substitution and the corresponding anti-aggregatory activity against Aβ_42_ fibrils.

### 2.3. Congo Red Assay of Aβ_42_ Inhibition by Olive Biophenols

To further investigate the Aβ_42_ fibrils inhibitory activity of olive biophenols, we have examined their activity through Congo red (CR) assay. CR is commonly used as histological dye in amyloid staining having linear and amphiphilic molecule striking spectrophotometric properties. The mechanism of CR binding with Aβ_42_ is still unclear, however a few studies have suggested the binding proceeds through the formation by both hydrophobic and hydrophilic interactions [30]. The Aβ_42_ fibrils inhibitory effect of olive biophenols in CR assay is almost similar and consistent with previous ThT assay. The reference inhibitor NDGA inhibited Aβ_42_ (IC_50_: 14.4 µM) almost in a similar potency as investigated in ThT assay (Figure 3A). The non-flavonoid olive biophenols, OL showed highest Aβ_42_ inhibition of 65% (IC_50_: 36.5 µM), followed by VB of 57% (IC_50_: 59.6 µM) and HT of 50% (IC_50_: 97.8 µM) respectively (Figure 3A). The least active CA inhibited Aβ_42_ by 47% at the maximum used concentration in the study and unable to achieve IC_50_ value. The flavonoids olive biophenols, LU showed the strongest inhibition of 61% (IC_50_: 46.3 µM) followed by QU of 55% (IC_50_: 73.8 µM) against Aβ_42_ fibrils, however RU inhibited Aβ_42_ fibrils by 48% at the maximum used concentration in the study and unable to achieve IC_50_ value (Figure 3B). The extracts olive biophenols, HTE showed highest inhibition of 69% (IC_50_: 28.4 µg/mL) followed by inhibition from OLE of 65% (IC_50_: 41.1 µg/mL) and OFE of 53% (IC_50_: 80.9 µg/mL), while OLP showed inhibition of 47% at the maximum used concentration in the study and unable to achieve IC_50_ value (Figure 3C).

A few studies have suggested that CR may act as a weak Aβ aggregation inhibitor and reduces neurotoxicity [31], whereas ThT is not known to inhibit fibril formation. However, we didn’t find inhibition performed by CR alone against Aβ_42_ fibrils (data not shown). The results of our study suggested that olive biophenols effectively inhibit the Aβ_42_ fibrils formation or elongations as well as able to disaggregates the formed Aβ_42_ fibrils, supporting the rationale for therapeutic use and future clinical studies towards the prevention or treatment of AD.

### 2.4. Neuroprotective Effects of Olive Biophenols against Aβ_42_ Induced Neurotoxicity in SH-SY5Y Cells

**Figure 4:**
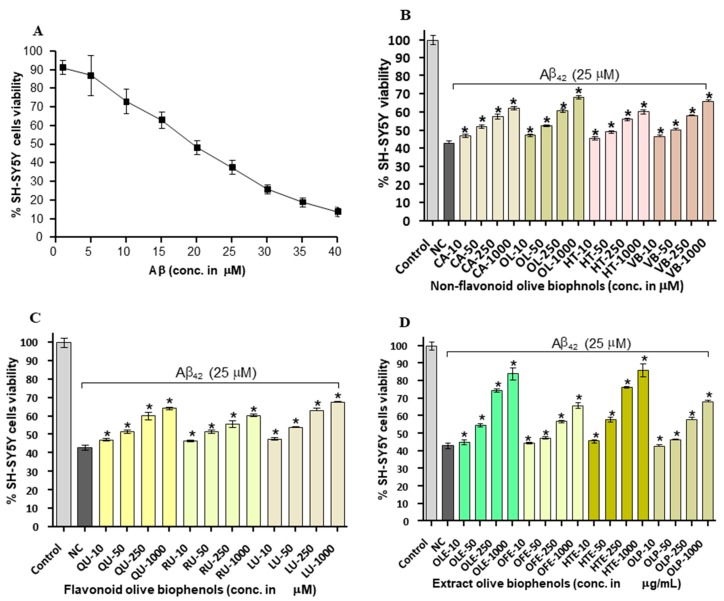
Aβ_42_ induced SH-SY5Y cells toxicity and protection by pre-incubation of olive biophenols for 24 h: (**A**) SH-SY5Y cells were treated with different concentrations of Aβ_42_ without olive biophenols for 24 h. SH-SY5Y cells were pre-incubated with different concentrations of (**B**) non-flavonoid olive biophenols, (**C**) flavonoid olive biophenols and (**D**) extract olive biophenols for 24 h followed by 25 μM of Aβ_42_ for 24 h. The results are mean ± SE of each parallel measurements analyzed by one-way ANOVA (*Tukey’s test*), * *p* < 0.001 *vs* negative control. NC: negative control (cells with Aβ_42_ without biophenols), CA: caffeic acid, OL: oleuropein, HT: hydroxytyrosol, VB: verbascoside, QU: quercetin, RU: rutin, LU: luteolin, OLE: olive leaf extract, OFE: olive fruit extract, HTE: Hydroxytyrosol extreme, OLP: Olivenol plus.

To investigate whether olive biophenols can rescue the cells suffered from Aβ_42_-induced toxicity, we investigated the viability of SH-SY5Y cells pre-incubated with olive biophenols. The Aβ_42_ produced neurotoxicity by 86% at the maximum concentration of 40 µM in the SH-SY5Y (Figure 4A), and achieved the LD_50_ at 20 µM. Our data supported the past *in vitro* studies having reported that the 20 µM of Aβ_42_ is enough to produce a substantial neurotoxicity in SH-SY5Y cells [28,32]. Pre-treatment (0–1000 μM) with olive biophenols, OL and VB were resulted in a significant increase in SH-SY5Y cells viability by 68% and 66% (*p* < 0.001) against Aβ_42_-induced neuronal death (Figure 4B), when compared to the control. Significant protective actions were also observed for CA and HT by 62% and 60% (*p* < 0.001) against the Aβ_42_-induced neurotoxicity in SH-SY5Y cells (Figure 4B). The highest preventative activity was shown by LU by 65% (*p* < 0.001) followed by QU (63%) and RU (59%) among the flavonoid olive biophenols (Figure 4C). Among the extract biophenols, HTE showed the strongest neuroprotection by 86%, followed by OLE having almost similar activity by 84% (*p* < 0.001), while OLP and OFE were protected SH-SY5Y cells by 68% and 66% (*p* < 0.001) against the Aβ_42_-induced neurotoxicity (Figure 4D).

Our results have showed that extracts olive biophenols as have great neuroprotective potential followed by the non-flavonoid and flavonoid olive biophenols against the Aβ_42_-induced neurotoxicity in the SH-SY5Y cells (Table 2).

### 2.5. Neuroprotective Effect of Olive Biophenols against Copper-Amyloid Induced Neurotoxicity in SH-SY5Y Cells

**Figure 5:**
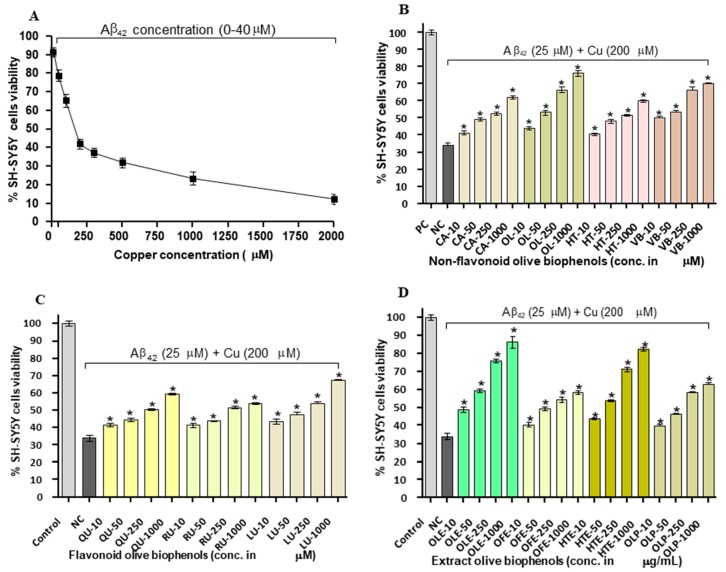
Copper-amyloid (Cu-Aβ_42_) induced SH-SY5Y cells toxicity and protection by pre-incubation of olive biophenols for 24 h: (**A**) SH-SY5Y cells were treated with 20 µM of Aβ_42_ along with different concentrations of copper for 24 h. The SH-SY5Y cells were pre-incubated with various concentration of olive biophenols and treated with 20 µM of Aβ_42_ and 200 µM of Cu (**B**) non-flavonoids olive biophenols, (**C**) flavonoid olive biophenols and (**D**) extract olive biophenols for 24 h followed by 25 µM of Aβ_42_ and 200 µM of copper for 24 h. The results are mean ± S.E. of each parallel measurements analysed by one-way ANOVA (*Tukey’s test)*, * *p* < 0.001 vs. negative control. NS: non-significant. C: positive control (cells with media), NC: negative control (cells with Cu-Aβ_42_ without biophenols), CA: caffeic acid, OL: oleuropein, HT: hydroxytyrosol, VB: verbascoside, QU: quercetin, RU: rutin, LU: luteolin, OLE: olive leaf extract, OFE: olive fruit extract, HTE: Hydroxytyrosol extreme, OLP: Olivenol plus.

To demonstrate how the transition metals (Cu and Fe) play a major role in the Aβ_42_ toxicity, we investigated the neuroprotective potential of olive biophenols against the Cu-Aβ_42_ induced neurotoxicity in SH-SY5Y cells. Exposure of SH-SY5Y cells to Cu-Aβ_42_ combination resulted in significant higher neurotoxicity (LD_50_: 17.6 µM) by 66% than the toxicity caused by Aβ_42_ (LD_50_: 20 µM) by 63% alone in the SH-SY5Y cells (Figure 5A). Treatment of the cells with non-flavonoid olive biophenols, OL to Cu-Aβ_42_ showed the highest neuroprotective activity by 76% (*p* < 0.001) (Figure 5B). Similarly, VB was the second highest neuroprotective agent by 70% (*p* < 0.001), followed by CA (62%) and HT (60%) (*p* < 0.001) were showed almost similar protective abilities respectively (Figure 5B). Moreover, the olive flavonoids, LU protected in the strength by 67% (*p* < 0.001) the SH-SY5Y cells followed by QU of 60% and RU of 54% (*p* < 0.001) against the Cu-Aβ_42_ induced neurotoxicity (Figure 5C). Olive extracts showed significantly higher neuroprotective activity at all concentrations tested (*p* < 0.001), where OLE was the most protective compound showed 87% of activity (Figure 5D). The second most protective compound, HTE protected by 82%, while OLP showed only 63% of protection followed by the least active OFE of 58% activity (Figure 5D).

Studies have suggested that copper ions have been found to accumulate in Aβ plaques and promotes their aggregation, play an important role in the generation of ROS and more specifically in hydrogen peroxide, which ultimately a risk factor for AD [33,34].

Our results suggested that extracts olive biophenols are superior to the olive non-flavonoids and flavonoids biophenols in terms of the presence of major phenolic compound oleuropein and hydroxytyrosol to protect the SH-SY5Y cells against Cu-Aβ_42_-induced toxicity, which could be a promising compound in AD therapy. However, the poor activities of flavonoids olive biophenols against Cu-Aβ_42_-induced toxicity are susceptible due to auto-oxidation and conversion into their *O*-methylated to their corresponding metabolites, lacking of a hydroxyl group ultimately leads to fewer neuroprotective activity in the SH-SY5Y cells [35].

The Aβ peptide contains metal-ion-binding sites which may provide a very promising target for the development of new therapeutics. In the present study, copper-treated SH-SY5Y cells may represent copper-overload model in the brain leading to the neurodegeneration in AD. The ongoing research development against metal-induced AD using Cu-specific chelating agents, chaperones, or antioxidants are focused in the prevention and treatment of Aβ neurotoxicity [36].

### 2.6. Neuroprotective Effect of Olive Biophenols against l-DOPA-Amyloid-Induced Neurotoxicity in SH-SY5Y Cells

**Figure 6:**
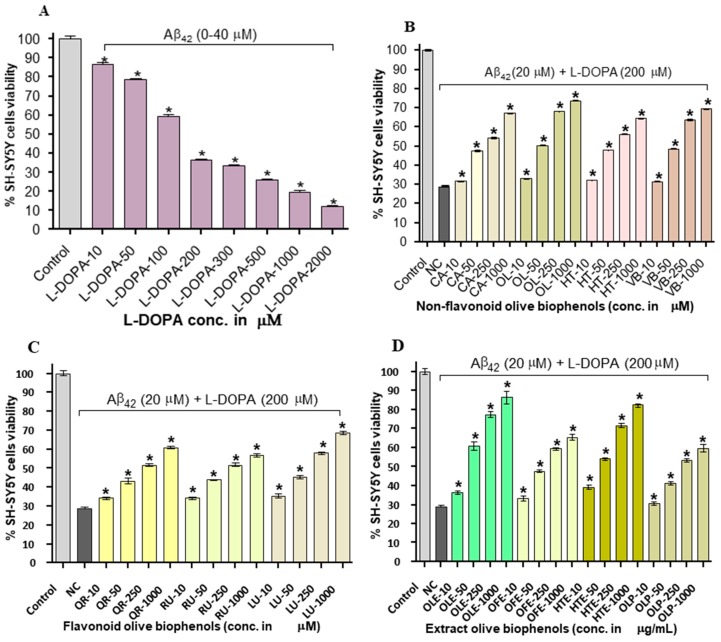
l-DOPA-amyloid (l-DOPA-Aβ_42_) induced SH-SY5Y cells toxicity and protection by pre-incubation of olive biophenols for 24 h: (**A**) SH-SY5Y cells were treated with 20 µM of Aβ_42_ along with different concentrations of l-DOPA for 24 h. The SH-SY5Y cells were pre-incubated with various concentration of olive biophenols and treated with 20 µM of Aβ_42_ and 200 µM of l-DOPA (**B**) non-flavonoids olive biophenols, (**C**) flavonoid olive biophenols and (**D**) extract olive biophenols for 24 h followed by addition of 25 µM of Aβ_42_ and 200 µM of l-DOPA for 24 h. The results are mean ± S.E. of each parallel measurements analysed by one-way ANOVA (*Tukey*’s test), * *p* < 0.001 vs. control. Control: cells with media, NC: negative control (cells with l-DOPA-Aβ_42_ without biophenols), CA: caffeic acid, OL: oleuropein, HT: hydroxytyrosol, VB: verbascoside, QU: quercetin, RU: rutin, LU: luteolin, OLE: olive leaf extract, OFE: olive fruit extract, HTE: Hydroxytyrosol extreme, OLP: Olivenol plus.

After SH-SY5Y cells were exposed to l-DOPA (0–2000 μM) and Aβ_42_ (0–40 μM) for 24 h caused significant dose dependent toxicity by 88% with LD_50_: 11.93 μM, which is almost two folds higher than the toxicity produced by individual Aβ_42_ (LD_50_: 20 µM), thus suggesting the synergistic action of toxicity (Figure 6A). Pre-treatment of non-flavonoid olive biophenol, OL showed the strongest protection by 74% (*p* < 0.001), followed by VB (69%), CA (67%) and HT (64%) against Aβ_42_- l-DOPA-induced toxicity in SH-SY5Y cells (Figure 6B). The flavonoid LU exhibited the strongest SH-SY5Y cells protection by 69%, followed by QU (61%) and RU by 57% (*p* < 0.001) (Figure 6C). The extracts olive biophenols showed markedly significant protection of SH-SY5Y cells at all concentrations tested (*p* < 0.001) against l-DOPA induced toxicity. A similar trend was observed for the extract OLE showed the highest protection of 86%, followed by HTE (82%), however OFE (65%) and OLP (60%) were showed the moderate protection of SH-SY5Y cells against Aβ_42_-l-DOPA-induced toxicity (Figure 6D). The possible mechanism of SH-SY5Y cells neurotoxicity through the production of reactive oxygen and nitrogen species (RONS), quinones and methylated l-DOPA products resulted from their metabolism and/or the auto-oxidation of l-DOPA [37]. Pre-treated SH-SY5Y cells with olive biophenols prevented overproduction of RONS and scavenge the excess RONS, may reduce the apoptosis by enhancing and prolonging the up-regulation of the survival pathways including phosphatidylinositol-3-OH kinase/AKT and JNK (c-Jun N-terminal kinase). Olive biophenols reduced the l-DOPA toxicity and protected the SH-SY5Y cells, could be a promising neuroprotective compound against neurodegenerative diseases such as Parkinson’s or AD.

### 2.7. Behavioural Analysis

#### 2.7.1. Light and Dark Test

The total time spent in the dark side have been determined and does not showed significant difference between the olive leaf extract fed and normal diet fed in both the group, wild and APPswe mice. The results showed that olive fed diet group mice either wild (*p* < 0.23) or APPswe/PS1dE9 (*p* < 0.321), both spent slight less time in the dark side compare to the normal diet fed group. A few studies have suggested that light and dark test effect is only observed in certain strains of mice or with certain drugs [38]. 

#### 2.7.2. Novel Object Recognition

At the end of diet treatment with or without OLE, novel object recognition test results revealed that no significant differences were found in the total amount of exploration time between the APPswe/PS1dE9 and wild mice treated with OLE and normal diet. However, the exploration mean time for APPswe/PS1dE9 mice fed on normal diet was slightly higher than the familiar object exploration (*p* = 0.969). Moreover, APPswe/PS1dE9 mice on olive extract diet does not showed any significant (*p* = 0.952) improvement in exploration of object compare to the normal diet group. The results suggested that at the end of study, APPswe/PS1dE9 mice at the age of 23 weeks were unable to develop spontaneous behaviour change and can’t interact more with a novel object than with a familiar one.

#### 2.7.3. Barnes Maze Test

The Barnes maze was divided into few quadrants such as target, east, south and opposite. The primary goal of each test animal was to reach the target quadrant in the proper time, if they failed then their scored would be zero. The results were showed no significant difference to reach the target quadrant as well as other quadrant between the normal diet and OLE diet fed wild mice. The APPswe/PS1dE9 group of mice group doesn’t showed significant difference found between the OLE and normal diet fed group. However, there was slight improvement in time to reach the east, south and opposite quadrant, shown by OLE diet fed APPswe/PS1dE9 mice, which was statistically non-significant. This finding suggested that 23-weeks old APPswe/PS1dE9 transgenic mice were not substantially influenced by learning and remembering locations or impaired in terms of long-term memory.

### 2.8. Amyloid Plaque Burden

**Figure 7:**
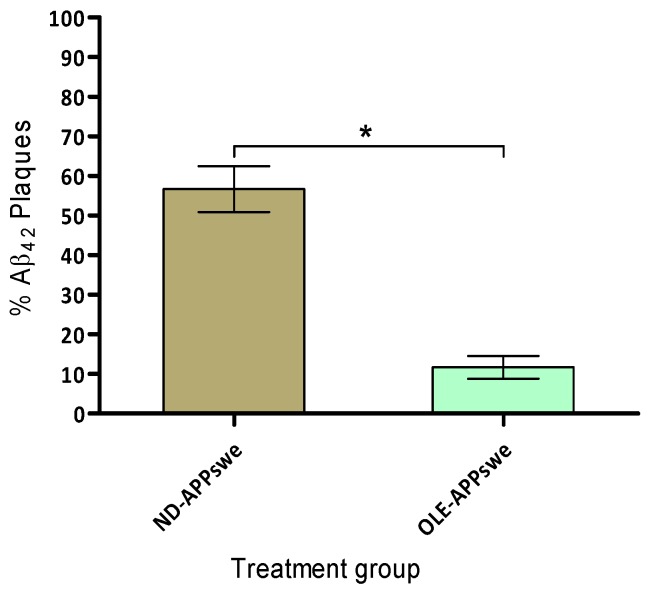
Amyloid plaque burden and olive biophenols protection. The result was analysed by one-way ANOVA, * *p* < 0.001.

We have investigated the amyloid plaque burden in the cortex and hippocampus of 6-months old APPswe/PS1dE9 mice treated with or without OLE for the 4 months. The quantitative analysis of total number of Aβ plaques in the specific area of the cortex and hippocampus revealed that the effect of OLE treatment was significant (*p* < 0.001) in the APPswe/PS1dE9 mice (Figure 7). The morphological characteristics of Aβ plaques load showed the largest plaque in the cortex and hippocampus area ranging between 1200–1600 µm^2^ respectively, which was markedly reduced in the brains of APPswe/PS1dE9 mice fed with OLE. 

The results demonstrated that concomitant OLE treatment to the APPswe/PS1dE9 mice cause significant improvement in amelioration of hippocampal neuropathological aspects and leading to reduce the amyloid plaque burden. An earlier study on olive leaf extract diet fed to TgCRND8 mice, showed improvement in behaviour with a significant reduction in Aβ levels [39].

### 2.9. Biochemical Analysis

#### 2.9.1. Serum Cholesterol Level

The results showed that there was no significant difference of cholesterol level between the OLE fed diet and normal diet in wild mice. While there was slight decrease in the serum cholesterol level (1.65 ± 0.36 mmol/L) amongst OLE fed APPswe/PS1dE9 mice from the normal fed diet APPswe/PS1dE9 mice (2.09 ± 0.56 mmol/L), but it was not statistically significant. A number of *in vitro* and *in vivo* studies suggested that the high intracellular cholesterol levels facilitate the processing of APP by β- and γ-secretase, thereby enhancing the release of Aβ [40,41,42]. In contrast, a few animal studies have suggested that total cholesterol levels were not significantly different between transgenic and wild-type mice during the development of AD neuropathology [43].

#### 2.9.2. Plasma Triglyceride Level

Elevated triglyceride levels have been reported in subjects with AD [44,45]. The present study showed no significant difference between the OLE fed diet and normal diet amongst APPswe/PS1dE9 and wild mice. While, there was a slight decrease in triglyceride level found (0.548 ± 0.07 mmol/L) in APPswe/PS1dE9 mice compare to normal diet fed APPswe/PS1dE9 (0.726 ± 0.26 mmol/L) controlled mice. However, an *in vivo* study has found no significant associations between plasma triglyceride in AD model of mice compared to controls [46].

#### 2.9.3. Plasma Glucose Level

Amyloid deposits are found in the pancreatic islets of most individuals with non-insulin-dependent diabetes mellitus [47]. The results showed that there was no significant difference between the OLE fed and normal diet fed APPswe/PS1dE9 mice. There was no any change found in wild mice fed with OLE and normal diet, while a slight decrease in plasma glucose level was found between APPswe/PS1dE9 OLE diet (12.72 ± 2.3 mmol/L) and APPswe/PS1dE9 normal fed diet 13.73 ± 2.75 mmol/L). 

## 3. Discussion

The present study provided the following three important findings. First, olive biophenols prevented in situ Aβ_42_ fibrilization and confirmed by electron microscopy, ThT assay and Congo red assay; second, olive biophenols showed a strong protective effect against Aβ_42_-induced cell death in human neuroblastoma SH-SY5Y cells, rescued the SH-SY5Y cells from Aβ_42_-induced cell death, Cu-Aβ_42_-induced cell death and Aβ_42_-l-DOPA-induced toxicities; and third, OLE effective in reducing Aβ neuropathology in AD mouse model (double transgenic APPswe/PS1dE9).

In this study, we used two ways to testify the Aβ_42_ inhibition, ThT and Congo red assay. Nonflavonoids biophenols, OL and VB were the leading potential direct Aβ_42_ fibrillization inhibitors might be due to the presence of C3 in OL [48], which is a unique site for antioxidant activity and its non-polar and non-covalent moiety interaction with the hydrophobic end of the Aβ fibril [49], and the presence of catechol moiety in VB [50]. However, the flavonoids olive biophenols were the intermediate Aβ_42_ fibrillization inhibitors, where LU was the strongest inhibitor over QU might be due to the presence of a C2-C3 double bond on the C-ring and possession of both a catechol group in the B-ring and the 3-hydroxyl group [51]. It has been suggested that the presence of 3-hydroxy, 4-keto groups of QU are essential for inhibition of Aβ fibrils growth [52]. The deleterious effect of commercial olive extract HTE on Aβ fibrils could be due to the presence of two major biophenols hydroxytyrosol and oleuropein [20], which may further cause synergic action between the individual biophenolic components and believed to be acting through the intermolecular π-π stacking, therefore inhibiting the aggregation of Aβ fibrils. 

Human neuroblastoma SH-SY5Y cells line model system is widely used for investigating and assessing the neuroprotective effects of natural compounds against the neurodegenerative diseases models including AD, because of their differentiation into neuron like cells and consistent biochemical features of mature neurons along with axonal expression of mature tau protein isoforms [20,53]. Recently, we have reported the neuroprotective effect of seven individual olive biophenols and four commercial olive extracts at physiologically relevant conditions against H_2_O_2_-induced cell death model in human neuroblastoma SH-SY5Y cells and suggested that neuronal cell death due to excessive oxidative stress-induced toxicity was significantly suppressed by olive biophenols treatment [20]. In this study, we investigated the effects of olive biophenols on Aβ_42_-induced toxicity in SH-SY5Y cells by pre-incubation with biophenols during the aggregation process of Aβ_42_ in the presence or absence of reference inhibitor. Among the olive phenolic compounds, (non-flavonoids) OL and VB; (flavonoids) LU and QU; and (extracts) HTE and OLE were strongly reduced the cellular toxicities and rescue SH-SY5Y cells against Aβ aggregates. In a similar manner, we have recently reported the anti-amyloid effect of olive biophenols through the amyloidogenic pathway inhibition, where OL and VB were the strongest inhibitor of BACE-1 enzyme [21]. To the best of our knowledge, this study represents the first attempt to identify the major olive phenolic compound(s) responsible for the *in vitro* anti-amyloidogenic effects.

Studies have shown that senile plaques in the AD-affected brain have elevated concentrations of transition metals specifically Cu, Zn, and Fe, suggested their interactions with Aβ fibrils alter the aggregation [5,54]. Our results showed that copper accelerated the Aβ_42_ fibril formations and aggregation might be due to high binding affinity of Cu with Aβ_42_ [9,55], and produce higher toxicity than the toxicity produced by Aβ_42_ in the absence of Cu in SH-SY5Y cells. However, the exact mechanism of Cu-Aβ_42_ co-treatment toxicity is unknow while it is believed that the binding of Aβ to redox active metal copper may facilitate redox cycling and lead to produce the highly reactive hydroxyl radical (OH^•^) in SH-SY5Y cells [8], resulting in an oxidative stress environment [7,9]. Interestingly, the olive biophenols OL, LU and OLE extract are potential compounds which were showed higher neuroprotective potential than the corresponding non-flavonoids, flavonoids and extract olive biophenols against the Cu-Aβ_42_-induced toxicity in SH-SY5Y cells. Recently, we have reported the olive biophenols specially VB, QU and HTE extracts rescue SH-SY5Y cells against Cu-induced toxicity [20]. Thus, from our past and present reported results, olive biophenols have demonstrated the potential not only to counter the Aβ_42_ fibrillization but also metal-induced Aβ_42_ fibrillization and rescue the SH-SY5Y cells from their corresponding toxicity. 

Due to the prolong use of l-DOPA in the Parkinson’s disease (PD) patients may cause less responsive and evoke side effects, however few studies have shown the neurotoxic effect of l-DOPA at the high concentration through the ability to generate free radicals in the SH-SY5Y cells [37], as well as the presence of accumulated l-DOPA-containing wrongly synthesize proteins in the brain of l-DOPA-treated PD patients [56]. Dementia and extrapyramidal are combine signs present in both AD and PD and may produce various degrees of clinical overlap between the two disease [57], thus we investigated the effect of olive biophenols against the l-DOPA-Aβ_42_-induced SH-SY5Y cells toxicity. Our results suggested that l-DOPA co-treatment with Aβ_42_ produces higher toxicity than the toxicity exhibited by l-DOPA alone in SH-SY5Y cells [20]. In addition, OL, LU and extract OLE were the strongest neuroprotective biophenols against the l-DOPA-Aβ_42_-induced toxicity in SH-SY5Y cells and suggested their mechanism of action through the free radical scavenging and direct Aβ_42_ fibrils inhibition [20]. 

Finally, we have investigated the effect of 4-months administration of OLE (50 mg/kg) on amyloid pathology along with the behavioural changes in the APPswe/PS1dE9 mice. Our results demonstrated that OLE significantly (*p* < 0.001) reduces the Aβ plaques in OLE fed APPswe/PS1dE9 mice compared to the control group and suggested that APPswe/PS1dE9 mice may exhibit fast amassing of Aβ inside the hippocampus beginning at approximately the age of 3-months prior to cognitive impairment [58]. Since, the extract OLE containing oleuropein as major biophenol, therefore we may suggest that oleuropein can cross the BBB and inhibit the production of Aβ fibrils and also disrupt the formed fibrils. Altogether, this strongly suggests that olive biophenols specially oleuropein can cross the BBB *in vivo*, and therefore have the potential to act centrally. Unfortunately, none of our behavioural analysis tests including NOR, light and dark test, and Barnes maze tasks were significant and demonstrated that the mice were unable to develop the cognitive deficits behavior in 4 months. Our non-significant behavioural analysis results raised interesting question as to whether change in behavioural develop before the amyloidosis or after the amyloidosis, however from the results suggested that amyloidosis develop since early age while, the behavioural aspect may change after ageing. A few studies have suggested that certain strain of transgenic mice showed increase in parenchymal Aβ load with Aβ plaques start from the age of four months, glial activation, and deficits in cognitive functions at the age of 6 months demonstrated by radial arm water maze and at 12–13 months seen with Morris Water Maze test [59]. In addition, it may depend on the type of transgenic mice strain which cause early or late behaviour changes. A few earlier studies have reported that APPswe PS1dE9 mice do not perform all cognitive tasks than the mice from all other genotypes and showed mild decreases in cholinergic markers [60]. In summary, we may suggest that APPswe/PS1dE9 mice have develop Aβ pathology earlier than the change is behavioural aspects, therefore the longer duration of study (>12 months) should be warrant for the evaluation of behavioural and biochemical changes. 

Taken together, we proposed the mechanism of Aβ aggregation inhibition by olive biophenols through the breakdown of the formed fibrils and interfere with the colloidal properties of aggregation rates and conformational preference of Aβ, ultimately leading to cause further inhibition of aggregation. In addition, hydrophobic attraction and conformational preferences of Aβ in the presence of olive biophenols were supposed to be identified as major determinants of their mechanism of interaction [61]. Due the presence of catechol moiety along with hydroxyl groups (ranging from 1–4), serves effective electron and hydrogen atom donors to neutralize free radicals and other reactive oxygen and nitrogen species (RONS), make olive biophenols an ideal candidate for targeting Aβ_42_.

This study is the first, to the best of our knowledge, to report the protective and comparative effects of seven individual olive biophenols and four olive extracts against Aβ toxicity and plaques load, rendering olive biophenols a promising compound to treat or prevent AD.

## 4. Material and Methods

### 4.1. Chemicals and Reagents

Oleuropein (OL), hydroxytyrosol (HT), luteolin (LU) and vebascoside (VB) were purchased from Extrasynthese, Genay Cedex, France. The four commercial preparations were purchased, *viz.*, Olive Leaf Extract^TM^ (OLE), equivalent to fresh leaf 1 g/mL or oleuropein 4.4 mg/mL from Comvita^TM^ (Brisbane, Australia); Olive Fruit Extract^TM^ (OFE), each mL stated to contain 5 mg of oleuropein, from Nature Goodness^TM^ (Smeaton Grange, Australia); Hydroxytyrosol Extreme^TM^ (HTE), each 100 mg olive leaf extract capsule stated to provide 25 mg of hydroxytyrosol, from ProHealth^®^ (Carpinteria, CA, USA); and 200 mg of Olivenol Plus^TM^ capsules (OLP), made with 12 mg (6%) of HIDROX^®^, a patented formula of HT derived from olive juice, from CREAGRI^TM^ (Hayward, CA, USA). Human amyloid beta (Aβ_42_) was purchased from APExBIO (Batch No.1), USA. Caffeic acid (CA), quercetin (QU), rutin (RU), dimethyl sulfoxide (DMSO), Tris-HCl buffer, copper chloride (CuCl_2_), neuroblastoma cell line (SH-SY5Y), dulbecco’s modified eagle medium (DMEM), fetal calf serum (FCS), 3-(4,5-dimethylthiazol-2-yl)-2,5-diphenyl-tetrazolium bromide (MTT), Thioflavin-T (ThT), and Congo red (CR) were purchased from Sigma-Aldrich, Castle Hill NSW, Australia. Nordihydroguaiaretic acid (NDGA) was purchased from Santa Cruz Biotechnology, USA. The plasma cholesterol, triglyceride and glucose kits (Lot. No. V42099; 982620 and 024201) were purchased from Thermo Scientific, Australia.

### 4.2. Sample Preparation

We have investigated and published the phenolic composition along with antioxidant activities of all the commercial olive extracts were assessed by HPLC-DAD, online-ABTS scavenging activity chromatograms and confirmed by LC-MS [20]. We found the presence of hydroxytyrosol and verbascoside olive biophenols in all the four commercial extracts (OLE, OFE, HTE and OLP) and demonstrated the strong online-ABTS scavenging activity [20]. However, oleuropein aglycone-1 and Luteolin-7-*O*-glucoside were detected in three extracts OLE, HTE and OLP, and OLE, OFE, HTE with the variable amounts [20]. The biophenol oleuropein was detected as the major phenolic constituents of the OLE and OFE extracts [20]. According to the manufacturers claim, OLE and HTE extracts were prepared from olive leaf, while OFE and OLP extracts were prepared from olive fruit pulp. Moreover, they suggested oleuropein as major constituent present in OLE (4.4 mg/mL) and OFE (5 mg/mL) extracts, while hydroxytyrosol was the primary phenolic constituents present in HTE (25 mg/100 mg extract) and OLP (12 mg/capsule) extracts. Our results were in the line of manufacturers’ preparation supported their phenolic constituents’ claims.

All the standard non-flavonoid biophenols (CA, HT, OL and VB), flavonoid biophenols (LU, QU and RU) and the commercial olive extracts (OLE, OFE, OLP and HTE) were prepared in 50% methanol, followed by ultra-sonication and filtration (nylon syringe filter 0.25 μm) before each assay, and consumed within 4 h of preparation to minimise air-oxidation. 

### 4.3. Aβ_42_ Fibril Preparation and Aggregation Inhibitory Assay

The stock solution of 3.5 mM Aβ_42_ was prepared by dissolving the lyophilized peptides in 10% DMSO followed by vortexing and sonification, and stored immediately at −80 °C. Twenty μL of Aβ_42_ (50 μM in 10 mM Tris-HCl buffer having pH 7.4) from the stock solution was incubated for 7 days to grow the fibrils at the room temperature without agitation.

#### 4.3.1. Transmission Electron Microscope (TEM) Imaging

The Aβ_42_ fibril imaging with or without olive biophenols was obtained by TEM described elsewhere [62]. Twenty μM of Aβ_42_ fibrils was incubated in the presence or absence of 0–2000 µM of each olive biophenols (OL, QU and OLE) for 24 h at 37 °C. Ten µL aliquot of each sample was spotted onto a glow-discharged, carbon-coated formvar grid and incubated for 30 min. The droplet then was displaced with an equal volume of 2.5% glutaraldehyde (*v*/*v*) and incubated for an additional 5 min. Finally, the grid was stained with 10 μL of 3% 0.22 µm filtered uranyl acetate (*v*/*v*) twice, and the solution was gently wicked off using Whatman’s grade-1 qualitative filter paper and the grid was then air-dried. Samples were examined by using a Hitachi H7100FA TEM (Hitachi, Japan) at the Centre for Advanced Microscopy, Australian National University, Canberra. All the images were captured at a voltage of 125 kV and an instrumental magnification of 2000×.

#### 4.3.2. Thioflavin-T (ThT) Fluorometric Assay

Thioflavin-T (ThT) assay was performed according to the method described elsewhere [26] with slight modification including the adjustment of volume and concentration to perform the assay in a microtiter plate. Five μM of ThT was prepared in Tris-HCl buffer pH 7.4 and stored in an aluminium foil wrapped vial to protect from the photo-oxidation. Nordihydroguaiaretic acid (NDGA) was used (50 µM) as reference inhibitor [63], however 200 µL of Tris-HCl buffer pH 7.4 added with 20 µL of ThT and Aβ_42_ (50 µM) were used as control. The black sterile 96 microplates were then incubated with equal volume (20 µL) of different olive biophenols in a concentration range of 10–1000 µM/µg along with the pre-formed Aβ_42_ fibril for 2 h unshaken at the room temperature. The absorbance was measured at excitation 450 nm and emission 480 nm on Cary Eclipse Fluorescence Spectrophotometer (Agilent technologies, Mulgrave VIC, Australia). 

#### 4.3.3. Congo Red Binding Assay

Congo red (CR) binding with Aβ_42_ assay was assessed according to the previously described method [64], but with the adjusted volumes in a microtiter plate. Briefly, 225 µL of 20 µM Congo red in phosphate buffer saline (20 mM potassium phosphate, pH 7.4, containing 0.15 M sodium chloride) along with 25 µL of 50 µM fibrillized Aβ_42_ were used as control. The black sterile 96 microplates were then incubated with 25 µL of 50 µM of NDGA as reference fibrillization inhibitor or different concentration of olive biophenols ranging from 10–1000 µM/µg were along with the pre-formed Aβ_42_ fibril for 2 h unshaken at the room temperature. The absorbance of the resulting solutions was measured at excitation 480 nm and emission 540 nm using a Cary Eclipse Fluorescence Spectrophotometers (Agilent technologies, Australia). 

### 4.4. Cell Culture

Human neuroblastoma (SH-SY5Y) cells were cultured (manufacturer protocol) in 50% Minimum Essential Media (MEM) and 50% Ham’s F-12, and supplemented with 15% inactivated fetal calf serum, 1% of 100 units/mL penicillin/streptomycin, 1% l-glutamine and 1% NEAA under 5% CO_2_/95% humidified air at 37 °C in an incubator. The culture media was changed every two days followed by cells passage at 80–90% of confluency usually every third day using trypsin-EDTA solution. Hemocytometer was used for counting and differentiating the viable and dead cells by adding 10% Trypan Blue.

#### 4.4.1. Aβ_42_ Induced SH-SY5Y Cells Toxicity and Olive Biophenols Treatment

The SH-SY5Y cells (5 × 10^3^ cells/well) were seeded 24 h before the experiments in a clear sterile 96-well plate and grown in 95% humidified cell incubator at 37 °C under a 5% CO_2_ atmosphere. In order to determine the toxicity of Aβ_42_ in SH-SY5Y cells, different concentrations (0–40 µM) of Aβ_42_ fibrils (after 5 days of incubation at the room temperature) was treated with the SH-SY5Y cells. For neuroprotective effect, different concentrations (10–1000 μM/μg) of freshly prepared olive biophenols were incubated with SH-SY5Y cells (5 × 10^3^ cells/well) for 24 h followed by 25 μM of Aβ_42_ fibrils (showing 50–60% toxicity) treatment and incubated further for 24 h at 37 °C under 5% CO_2_/95% humidified air in an incubator.

#### 4.4.2. Aβ_42_-Copper Induced SH-SY5Y Cell Toxicity and Olive Biophenols Treatment

In order to determine the toxicity of Aβ_42_-Copper combination in the SH-SY5Y cells, various concentrations (0–2000 µM) of copper was added together with Aβ_42_ fibrils (0–40 μM) in eight equal divided doses to overcome the bias in the sterile clear 96-well plates containing SH-SY5Y cells (5 × 10^3^ cells/well) followed by incubation over 24 h at 37 °C under 5% CO_2_/95% humidified air. Freshly prepared olive biophenols in various concentrations (10–1000 μM/μg) were incubated with SH-SY5Y cells (5 × 10^3^ cells/well) for 24 h and maintained at 37 °C under 5% CO_2_/95% humidified air in an incubator. To investigate the neuroprotective effects of olive biophenols against Aβ_42_-copper-induced toxicity, the pre-treated SH-SY5Y cells with olive biophenols were allowed to expose with 25 μM of Aβ_42_ fibrils and 200 μM of copper (showing 60–70% toxicity) followed by incubation for 24 h.

#### 4.4.3. Aβ_42_-l-DOPA Induced SH-SY5Y Cell Toxicity and Olive Biophenols Treatment

In order to determine the toxicity of Aβ_42_-l-DOPA combination in the SH-SY5Y cells, freshly prepared l-DOPA in various concentrations (0–2000 μM) was incubated with (0–40 μM) Aβ_42_ fibrils in the sterile clear 96-well plates containing SH-SY5Y cells (5 × 10^3^ cells/well) followed by incubation over 24 h at 37 °C under 5% CO_2_/95% humidified air. Different concentration of freshly prepared olive biophenols (10–1000 μM/μg) were incubated with SH-SY5Y cells (5 × 10^3^ cells/well) for 24 h at 37 °C under 5% CO_2_/95% humidified air in an incubator. To access the neuroprotective effects of olive biophenols against Aβ_42_-l-DOPA-induced toxicity, the pre-treated SH-SY5Y cells were exposed to 25 μM of Aβ_42_ fibrils and 200 μM of l-DOPA (showing 60–70% toxicity) followed by further incubation for 24 h.

#### 4.4.4. Cell Viability Assay

Cell viability was determined by MTT assay based on reduction of MTT to insoluble formazan, the amount of produced formazan reflects the cell viability. The reaction mixture medium was replaced and treated with 10 μL of MTT (5 mg/mL) in phosphate buffered saline (pH 7.4) to the each well containing SH-SY5Y cells, olive biophenols, Aβ_42_/Aβ_42_-Copper/Aβ_42_-l-DOPA followed by incubation for 4 h at 37 °C [65]. The formazan crystals were generated by viable mitochondrial succinate dehydrogenase from MTT. The supernatant was then aspirated off and the formazan crystals were dissolved in 50 μL of DMSO. After 15 min of reaction time, the absorbance was measured at 570 nm using the Omega Star micro plate reader [66]. The experiments were performed in triplicate and the cells viability was expressed as percentages of survival relative to the control sample.

### 4.5. Animals and Ethical Considerations

**Figure 8:**
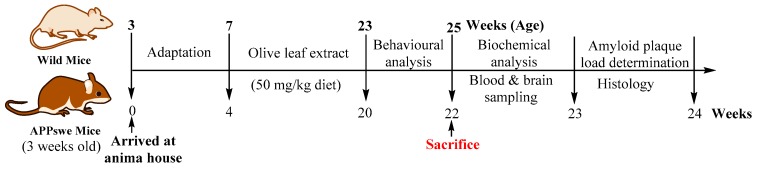
Schematic representation of the APPswe/PS1dE9 mice study schedule.

A total 30 (16 wild and 14 APPswe/PS1dE9) male mice of 3 weeks old age were received as a generous gift from University of Queensland, Australia, in-housed with food and water available *ad libitum* and maintained on a 12:12-h light/dark cycle with lights in a temperature-controlled (20 ± 2 °C) room prior to experimental manipulation at the animal house, School of Biomedical Sciences, Charles Sturt University. Age-matched non-transgenic litter-mate mice (WT) were used as controls. The APPswe mice (TG) overproduce human Aβ_40_ and Aβ_42_ peptides and develop progressive cerebral amyloid beta deposits and learning and memory impairment [57,67]. All the experimental procedures and protocols (Reference No. 12/006) were approved (23 December 2011) by the Animal Use Ethics Committee of Charles Sturt University, Australia (Figure 8).

### 4.6. Diet

The wild (WT) and APPswe (TG) mice were divided into the treatment and control group. The treatment group of mice were received 50 mg/kg of oleuropein containing olive leaf extract (OLE), while the control group were received normal pellets beginning at 7 weeks of age for the period of 4 months (Table 3). The olive biophenols dosage was chosen for the treatment group was based on equivalent doses used in studies that showed efficacy in animal models [39,68]. 

### 4.7. Experimental Procedures

The wild (WT) and transgenic mice (TG) were divided into groups: control group (*n* = 7; both wild and transgenic mice) and OLE group (*n* = 7; both wild and transgenic mice) were received the control diet and OLE diet for 16 weeks to establish the animal model of Alzheimer’s disease and effect of dietary pattern. The body weight and food intake of the mice were monitored every day. In order to evaluate the anxiety, spatial memory, and learning and memory tasks, both the control and OLE group mice were trained at the end of 23 weeks of age and prior to the experimental performance of the battery of behavioural tasks.

#### 4.7.1. Light and Dark Test

In the light and dark test, the distance travelled and time spent in a brightly illuminated, aversive test arena compared to a dark area are indicators of anxiety in rodents [69]. The test was conducted as previously described method with slight modification [70]. The apparatus consisted of a non-transparent polypropylene cage separated into two compartments by a partition having a small opening at floor level. The larger compartment was open topped, transparent, and brightly illuminated by white light from a 60 W desk lamp positioned above the light chamber. The smaller compartment was close-topped and painted black. Mice were individually placed in the centre of the light compartment, facing away from the partition and allowed to freely explore the apparatus for 10 min. The apparatus was cleaned with a 30% ethanol solution between each run of mouse. The number of light dark transitions between the two compartments and the total time spent in the dark compartment were automatically recorded via photocells located at the opening between compartments, connected to a data storage device.

#### 4.7.2. Novel Object Recognition Test

The Novel Object Recognition (NOR) investigate the spontaneous behaviour of animals that spend more time exploring a novel object compared with familiar object. NOR test was conducted according to the previously described method [71] with modification in a plexiglass box (25 cm × 25 cm × 25 cm) with evenly illuminated sound-proof box. The experimental procedure includes 4 phases: pre-habituation, habituation, training, and testing. On the day 1 of test, animals were allowed to explore the testing room 30 min before the experiment to familiarize with the environment followed by freely explore the box in the absence of objects for 5 min. The habituation of mice was conducted on the day 2 and 3 to the empty box for 20 min per day. The training trial followed by a testing trial conducted on day 4 for each mouse. Two identical objects were placed on the two opposite positions within the box at same distance from the nearest corner in the training trail. Mice were allowed to interact with the identical objects for the period of 10 min followed by returning to the home cages. Mice were placed back to the same box after an hour, where one of the two familiar objects were replaced with a novel one, to start a 5 min testing phase. In the present study, different shapes and colours (black and white) objects were used but identical in size. Their activities were recorded by an overhead video camera (BL-C131, Panasonic, Fukuoka, Japan) connected to a Windows PC, and horizontal locomotion and rearing scores were calculated by using any-maze software. 

#### 4.7.3. Barnes Maze Test

The Barnes maze test used to investigate spatial-learning task that allows mice to use spatial cues to locate a means of escape from a mildly aversive environment. The Barnes maze test was adopted from elsewhere with slight modification [72]. Barnes maze is a white acrylic circular 90 cm in diameter disk consisting 12 equally spaced holes (4 cm in diameter) located 5 cm from the edge. Each hole could be opened or closed by means of a sliding, white acrylic door. In addition, a black acrylic escape box (8 × 8 × 8 cm), to which the mice gained access by way of a ridged, white acrylic ramp (30 incline), could be fitted below any of the holes in place of the door.

The mice were interacted with the Barnes maze in three phases: habituation (1 day), training (2–4 days in the short or long training paradigms), and probe (1 day). Each trial started by placing a mouse inside the start box positioned centrally on the maze. Prior to the start of each experiment, mice were acclimated to the testing room for 60 min followed by a day of habituation to the tube leading to the home cage of the mouse. Each mouse was trained for 2–4 days (three daily trials, 180-s cut-off, intertrial interval of 15 min) to find the target hole among 12 identical holes. During the training phase, primary latency and primary hole search (HS) were investigated and recorded. On the probe day, escape cage was removed and the mice were placed inside the opaque cylinder in the center of the maze for 15 s followed by turning on the buzzer and the removal of cylinder. To explore the maze, each mouse was given 2 min, the buzzer was turned off at the end of test and the mouse was returned to their holding cage. Measurement of time spent per quadrant and HS per quadrant were recorded in the probe phase. Their behavioural activity was recorded by an overhead illuminated halogen light with video camera (BL-C131, Panasonic, Fukuoka, Japan) connected to a Windows PC, and horizontal locomotion and rearing scores were calculated by using any-maze software. 

#### 4.7.4. Blood Biochemistry

Blood samples were collected from the retro-orbital plexus of mice under phenobarbital anaesthesia condition. The collected blood samples in the Eppendorf tube were subjected to immediate centrifugation (3000× *g*) in Eppendorf centrifuge 5424R for 10 min at 4 °C. The plasma was collected and stored at −80 °C. The plasma cholesterol, triglyceride and glucose were determined by using commercially available kits. Briefly, 300 μL of reagent with 3 μL distilled water gives the blank well, while 300 μL of reagent with 3 μL of calibrator gives the reading of standard. Three hundred μL of reagent with 3 μL of plasma gives the test measurement. The microplate was incubated for 10 min followed by reading on Versamax Tunable (Molecular Devices, Sunnyvale, CA, USA) automated microplate reader at 500 nm for plasma cholesterol and triglyceride determination, however plasma glucose was determined at 340 nm. The results were calculated as follows:

$$Cholesterol\;or\;Triglyceride\;or\;Glucose\;=\frac{change\;in\;absorbance/\min of\;unknown}{change\;in\;absorbance/\min of\;calibrator}\;\times calibrator\;value$$

#### 4.7.5. Assessment of Amyloid Plaque Burden

The mice were sacrificed after completion of the behavioural analysis by using lethal dose of pentobarbital, followed by removal of brain and sagittal division. For the protein analysis, cortical and hippocampal brain samples from one hemibrain of both control and OLE treated WT and TG mice were immediately sectioned, snap-frozen and stored at −80 °C. The rest of the hemibrain was postfixed in phosphate-buffered 4.0% paraformaldehyde, pH 7.4, at 4 °C for 48 h, rinsed in PBS and paraffin embedded for Congo red staining.

After mounting the brain with the wax, the hippocampal specified samples were subjected to microtome and slicing in 10 μ thickness film and prepared the slides in triplicate on water bath. After air drying the slides were subjected to Congo red (0.5% in 80% of ethanol and sonificated 15 min followed by Whatman’s paper filtration) staining followed by mounting and fixed by cover slit. Each slide was encoded with related animal code and randomized blindly for the microscopy. The amyloid plaques were counted in specific area of specimen slides in triplicate.

### 4.8. Statistical Analysis

Statistical significance was evaluated by One-way analysis of variance (ANOVA), IC_50_ and *Tukey’s tests* using GraphPad Prism 5.0. Statistical significance of the animal behaviour was analysed by two ways ANOVA using ANY-maze software and SPSS software (version 14.0; SPSS for Windows, Chicago, IL, USA). All the experiments were performed in triplicate, and the data are presented as mean ± standard deviation (SD) with the significant difference at the level of *p* < 0.001.

## 5. Conclusions

Inhibition of amyloid formation and disruption of the formed fibrillar assemblies are still one of the major therapeutic strategies proposed for the prevention and treatment of AD or amyloid-related diseases. Olive biophenols, OL and HT represent the molecules of major interest for their biological and pharmacological properties, and with no doubt, are among the most investigated antioxidant natural compounds. In addition, olive biophenols VB and LU were also shown the consistent significant activity against Aβ fibrils. The amyloid and metal induced toxicities were rescued more prominently by olive extract OLE in SH-SY5Y cells, however HTE was the immediate successor. It is suggested that olive biophenols may act on Aβ fibrils aggregate mostly through aromatic and hydrophobic interactions. The anti-amyloid capacity of olive biophenols were suggested mainly depended on the catechol moiety, the number of hydroxyl groups and a double bond conjugated to the 4-position on the aromatic ring. The results also suggest the multiple mechanism of olive biophenols in anti-amyloidogenic including antioxidant, BACE-1 inhibition, and HDAC inhibition [21].

Taking these findings together, we propose oleuropein, verbascoside and rutin as therapeutic candidates for preventing AD. In addition, both extracts OLE and HTE could be suggested and suitable as functional food ingredient due to its stronger anti-amyloidogenic activity.

## Figures and Tables

**Table 1:** Amyloid fibrils (Aβ_42_) inhibition by olive biophenols.

Olive Biophenols		Thioflavin-T Assay		Congo-Red Assay	
		IC_50_	% Inhibition	IC_50_	% Inhibition
Non-flavonoids	Nordihroguaretic acid (NDGA)	15.4 µM	70 ± 0.5	14.4 µM	69 ± 0.4
	Caffeic acid (CA)	ND	46 ± 0.32	ND	47 ± 0.31
	Hydroxytyrosol (HT)	ND	45 ± 0.47	97.8 µM	50 ± 0.4
	Oleuropein (OL)	22.9 µM	61 ± 0.33	36.5 µM	65 ± 0.3
	Verbascoside (VB)	22.6 µM	61 ± 0.35	59.6 µM	57 ± 0.51
Flavonoids	Luteolin (LU)	36.9 µM	64 ± 0.4	46.3 µM	61 ± 0.33
	Quercetin (QU)	45.9 µM	57 ± 0.34	73.8 µM	55 ± 0.71
	Rutin (RU)	ND	49 ± 0.25	ND	48 ± 0.33
Extracts	Olive leaf extract (OLE)	45 µg/mL	60 ± 0.36	41.1 µg/mL	65 ± 0.4
	Olive fruit extract (OFE)	95.9 µg/mL	50 ± 0.43	80.9 µg/mL	53 ± 0.51
	Hydroxytyrosol extreme (HTE)	30.4 µg/mL	64 ± 0.34	28.4 µg/mL	69 ± 0.42
	Olivenol plus (OLP)	ND	ND	ND	ND

ND: Not detected, % inhibition: The percentage inhibitory activity was showed with the highest concentration (1000 µM standard and 1000 µg/mL extract) of each biophenols in the study.

**Table 2:** Neuroprotective effect of olive biophenols against Aβ_42_, Aβ_42_-Cu and Aβ_42_-l-DOPA induced toxicities in SH-SY5Y cells.

Olive Biophenols		Aβ-SH-SY5Y Toxicity	Aβ-Cu-SH-SY5Y Toxicity	Aβ-l-DOPA-SH-SY5Y Toxicity
		% Viability	% Viability	% Viability
Non-flavonoids	Control (SH-SY5Y-media)	100 ± 1.21	100 ± 1.13	100 ± 0.92
	Negative control	37 ± 1.41	34 ± 1.53	12 ± 0.37
	Caffeic acid (CA)	62 ± 0.53	62 ± 0.93	67 ± 0.43
	Hydroxytyrosol (HT)	60 ± 1.00	60 ± 0.84	64 ± 1.02
	Oleuropein (OL)	68 ± 0.69	76 ± 1.61	74 ± 0.23
	Verbascoside (VB)	66 ± 1.11	70 ± 0.48	69 ± 0.66
Flavonoids	Luteolin (LU)	65 ± 0.39	67 ± 0.52	69 ± 0.87
	Quercetin (QU)	63 ± 0.29	60 ± 0.52	61 ± 0.73
	Rutin (RU)	59 ± 0.59	54 ± 0.71	57 ± 0.85
Extracts	Olive leaf extract (OLE)	84 ± 3.17	87 ± 3.2	86 ± 3.2
	Olive fruit extract (OFE)	68 ± 1.31	58 ± 0.69	65 ± 1.49
	Hydroxytyrosol extreme (HTE)	86 ± 3.6	82 ± 0.91	82 ± 0.96
	Olivenol plus (OLP)	68 ± 0.74	63 ± 0.96	60 ± 2.43

Note: % viability: The percentage viability of the cells were showed by using the highest concentration (1000 µM standard and 1000 µg/mL extract) of each biophenols.

**Table 3:** Dietary schedule for the wild and transgenic APPswe/PS1dE9 mice.

Animals	Normal Diet	OLE Diet
Wild mice (Control)	Yes	No
Transgenic Mice (Control)	Yes	No
Wild Mice (OLE)	No	Yes
Transgenic Mice (OLE)	No	Yes
